# Ion Channel and Ubiquitin Differential Expression during Erythromycin-Induced Anhidrosis in Foals

**DOI:** 10.3390/ani11123379

**Published:** 2021-11-25

**Authors:** Laura Patterson Rosa, Martha F. Mallicote, Robert J. MacKay, Samantha A. Brooks

**Affiliations:** 1 Department of Animal Sciences, UF Genetics Institute, University of Florida, Gainesville, FL 32611, USA; lpattersonr@ufl.edu; 2 Etalon Diagnostics, Menlo Park, CA 94025, USA; 3 Department of Large Animal Clinical Sciences, College of Veterinary Medicine, University of Florida, Gainesville, FL 32608, USA; mallicotem@ufl.edu (M.F.M.); mackayr@ufl.edu (R.J.M.)

**Keywords:** non-sweater, rhodococcosis, chronic idiopathic anhidrosis, channelopathy, transcriptomics, RNA-seq, neonate

## Abstract

**Simple Summary:**

Macrolide treatment for *Rhodococcus equi* infections can result in severe adverse effects including hyperthermia and temporary anhidrosis. Despite these potentially lethal side effects, and a lack of understanding of the mechanisms causing them, macrolide drugs remain the recommended treatment for *R. equi* infections in foals. To better understand the molecular biology behind these adverse effects, we performed a differential gene expression study of skin biopsies of six healthy macrolide-treated foals experiencing anhidrosis. In total, 132 transcripts were significantly differentially expressed, and these genes belonged to functional ontologies relevant to sweat function. Genes involved in ubiquitination and ion-channel function were upregulated during the anhidrotic timepoint. These biological mechanisms play an important role in equine idiopathic anhidrosis and sweat gland function and warrant further investigation as potential targets for avoiding macrolide-induced temporary anhidrosis.

**Abstract:**

Macrolide drugs are the treatment of choice for *Rhodococcus equi* infections, despite severe side-effects temporary anhidrosis as a. To better understand the molecular biology leading to macrolide induced anhidrosis, we performed skin biopsies and Quantitative Intradermal Terbutaline Sweat Tests (QITSTs) in six healthy pony-cross foals for three different timepoints during erythromycin administration—pre-treatment (baseline), during anhidrosis and post-recovery. RNA sequencing of biopsies followed by differential gene expression analysis compared both pre and post normal sweating timepoints to the erythromycin induced anhidrosis episode. After Bonferroni correction for multiple testing, 132 gene transcripts were significantly differentially expressed during the anhidrotic timepoint. Gene ontology analysis of the full differentially expressed gene set identified over-represented biological functions for ubiquitination and ion-channel function, both biologically relevant to sweat production. These same mechanisms were previously implicated in heritable equine idiopathic anhidrosis and sweat gland function and their involvement in macrolide-induced temporary anhidrosis warrants further investigation.

## 1. Introduction

A primary cause of pneumonia in foals under six months of age, the gram-positive facultative intracellular pathogen *Rhodococcus equi* (also known as *Prescottella equi* and *Rhodococcus hoagii*) can be cultured from the environment on nearly all horse farms, but incidence of clinical disease is variable [1,2,3]. *R. equi* survives and replicates in macrophages within abscesses where most antibiotics fail to penetrate [4]. Although many antimicrobial agents are effective against *R. equi* in vitro [5], this efficacy does not often translate to in vivo clinical applications [6]. With comparably superior results, the combination of the macrolide erythromycin with rifampin became the treatment of choice for *R. equi* pneumonia in the late 1980s and early 1990s [7]. Both erythromycin and rifampin are lipid-soluble compounds that concentrate within macrophages and neutrophils [8], enabling these drugs to penetrate caseous material and kill *R. equi*.

Although generally well tolerated by foals, erythromycin can have severe adverse effects. These usually occur during the first five days of treatment and include diarrhea (35.6%), hyperthermia (24.7%), and respiratory distress (15.1%) [9]. Hyperthermia occurs when normal thermoregulatory mechanisms are overwhelmed by excessive metabolic heat production, excessive environmental heat, or impaired dissipation of heat [10]. In a small herd study, treatment for pneumonia with erythromycin alone or in combination with rifampin resulted in 90% of foals developing hyperthermia, with rectal temperatures ranging from 39.6 to 44.4 °C (103.2 to 112 °F). The remaining 10% of the foals died, presumably of heat stroke [11]. High ambient temperature and exposure to direct sunlight also work as contributing environmental factors to hyperthermia [11]. Supporting the connection between erythromycin and hyperthermia, 4 out of 12 otherwise healthy foals developed hyperthermia, diarrhea, and lethargy during a second study examining the effect of erythromycin on bronchoalveolar lavage cells [12]. 

To dissipate excess body heat, horses rely predominantly on evaporation of sweat from the skin surface [13,14]. Hypohidrotic or temporarily anhidrotic horses suffer considerably, especially during warm seasons of the year, and have a reduced quality of life [15,16]. Clinical signs comprise partial or complete loss of sweat response leading to hyperthermia, reduced appetite and depression [17,18], and severe cases are may lead to convulsions and death, usually due to overheating [15,16]. Without sweating, estimates suggest that a horse galloping for 3.22 km (2 miles) could experience a core temperature increase of 6.1 °C (11 °F). 

Macrolide-associated hyperthermia, particularly during erythromycin treatment, is caused by a reduced ability to dissipate heat via evaporation of sweat [19]. Foals treated orally with erythromycin base (25 mg/kg TID) completely lost the sweat response to intradermal terbutaline within two days of treatment and did not fully recover for an average of 30 days [20]. Following cessation of the erythromycin treatment, treated foals became hyperthermic and tachypneic if exposed to hot and humid environmental conditions [19,20]. 

The connection between macrolide drugs and temporary anhidrosis in foals is well established, however the mechanisms leading to this potentially lethal side effect have yet to be elucidated. Differential gene expression of non-sweating versus normally sweating skin tissue provides a novel approach to macrolide-induced temporary anhidrosis. By leveraging transcriptome analysis of skin biopsies of foals treated with erythromycin, this study aimed to explore changes in gene expression during the pathogenesis of anhidrosis secondary to erythromycin administration.

## 2. Materials and Methods

### 2.1. Experimental Animals and Design

We utilized six pony-cross foals belonging to a research herd: two males and four females, aged between 26 to 72 days old and weighing between 29 kg to 136 kg. Sampling was performed by trained veterinarians, under the University of Florida Institutional Animal Care and Use Committee approved protocols. At enrollment, we performed a clinical evaluation of each foal including complete blood counts (CBC) to ensure that all were healthy enough to participate in the study [21]. 

**Figure 1:**
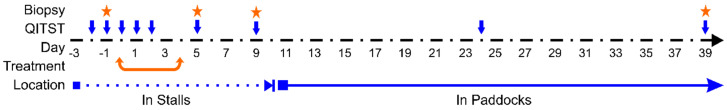
Timeline and Experimental Design. Stars represent biopsy days, and down arrows days when the quantitative intradermal terbutaline sweat test (QITST) was performed. Arrow range represents treatment days, going from day 0 to day 4.

**Figure 2:**
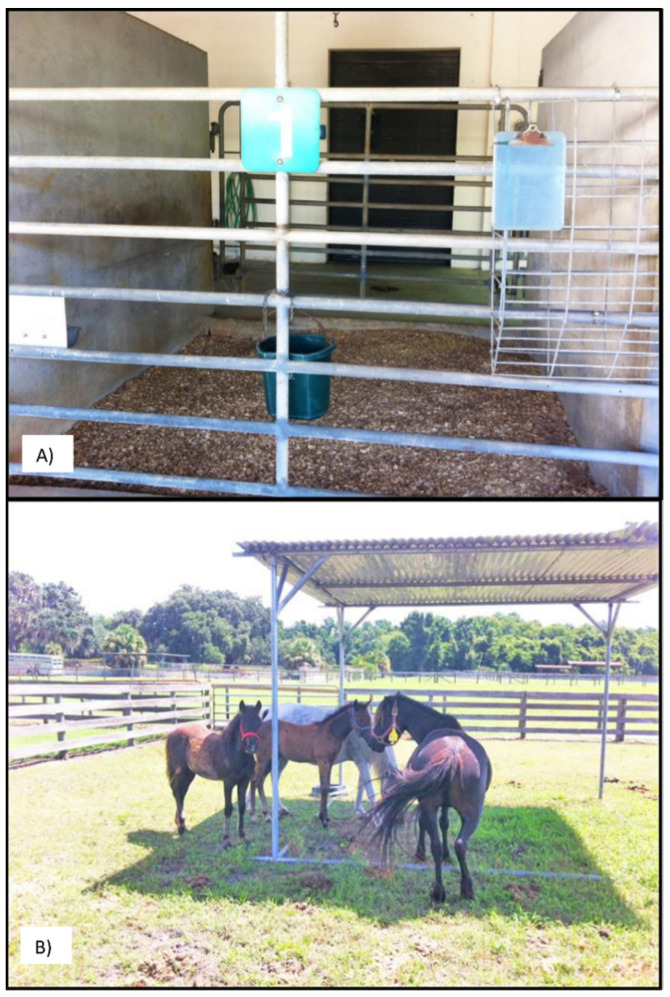
Locations where the study was conducted. (**A**) Open stalls and (**B**) Paddocks with covered area. Photo courtesy of Amy Stieler and Martha Mallicote.

The experiment was conducted between May and October 2015. The experimental period proceeded as following (Figure 1): day −3, foals and their dams brought into stalls for acclimatization; days −2, −1, 0—daily QITSTs to establish baseline sweat capacity; days 0 to 4 (beginning after last baseline QITST)—oral macrolide treatment (erythromycin); days 5 to 10—foals remain in stalls during period of hyperthermia risk; days 10 to 39—foals and their dams taken from stalls and kept in small pasture enclosures (Figure 2); days 1, 2, 5, 9, 24, 39—QITSTs to establish the quantitative profiles for induction of, and recovery from, anhidrosis. A physical examination including temperature, pulse rate, and respiratory rate was performed daily throughout the experiment early in the morning (6 to 8 AM). Temperatures were also checked in the evening (6 to 8 PM) and, on days 3 to 13, at midday (noon to 2 PM) to detect hyperthermia. Foals with rectal temperatures above 39.4 °C were hosed down and kept under fans until their temperature dropped below 38.9 °C. 

It was previously noted that environmental effects did not significantly affect sweat response for different macrolide treatments [19]. Still, to decrease the odds of heat shock, ambient environmental temperature and relative humidity data were collected each time the foals were restrained for measurement of rectal temperature. If the product of temperature × relative humidity exceeded 1400 °C% at a morning reading, then additional temperature monitoring took place during the day. For this study period, the recorded environmental average high temperature was of 32.5 °C (±4 °C) and average low temperature of 20.7 °C (±5.3 °C) (Florida Climate Center, Gainesville RGNL AP Station Data). If rectal temperatures exceeded 39.7 °C under these circumstances, foals were hosed down and returned to stalls under fans until their temperature dropped below 38.9 °C.

### 2.2. Erythromycin Treatment

Erythromycin base was prepared by a compounding pharmacy (Westlab Pharmacy Gainesville, FL, USA). A treatment coordinator mixed each powder with corn syrup in 35-mL dose syringes for each foal, with morning, midday, and evening labels. Treatment doses were calculated on foal weights recorded the day before each administration for 25 mg/kg, three times daily (TID). Erythromycin was administered orally to foals in the morning (6 to 8 AM), midday (noon to 2 PM) and evening (6 to 8 PM).

### 2.3. Quantitative Sweat Tests

**Figure 3:**
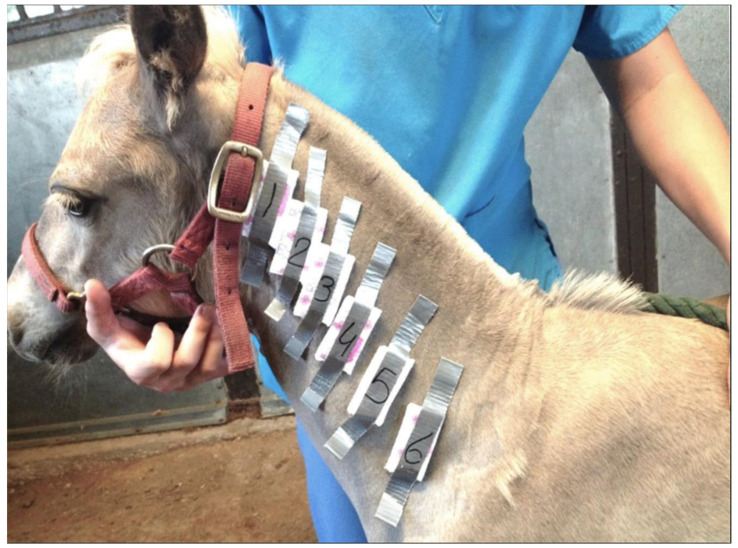
QITST testing on a study individual, with serial diluted terbutaline intradermal injection sites represented by numbered pads. Photo courtesy of Amy Stieler.

QITSTs were performed as previously described in MacKay et al. [22]. We alternated sides of the neck for successive QITSTs. In brief, foals and their dams were restrained without the use of sedatives and a 2-inch-wide by 12-inch-long area on the lateral neck clipped parallel to one inch below the dorsal margin of the foal’s neck. Starting at the cranial aspect of this strip, six 0.1 mL intradermal injections of serial 10-fold dilutions of the β-adrenergic agent, terbutaline sulfate in 0.9% saline, were administered through a 25-ga needle at approximately 1-inch intervals as follows: 0 (control), 1 × 10^−4^, 1 × 10^−3^, 1 × 10^−2^, 0.1 and 1 g/L. Pre-weighed individual sections of absorbent pad (3.2 × 6.4 cm) were secured with tape over each injection site (Figure 3). Thirty minutes later the pads were removed, sealed in plastic bags and reweighed on the same day. The absorbed sweat was quantified as weight change for each individual pad. QITST data were normalized through log transformation and interaction effects among treatment/day and terbutaline concentration were explored by 2-factor within-subjects repeated-measures general linear model procedures. For significant 2-factor (day × terbutaline concentration) interactions (i.e., *p* < 0.05), we performed post hoc pairwise comparisons using 2-tailed paired *t*-tests with Bonferroni corrections. All statistical calculations were performed using a commercial software package (PASW Statistics 18; SPSS Inc., Hong Kong). Significance in all analyses was ascribed only to *p* < 0.05.

### 2.4. Biopsy Collection

After desensitization of the neck area with lidocaine (not infiltrated under the biopsy site), we collected a local 8-mm skin punch biopsy on days −1, 5, and 39 (baseline, anhidrotic, and recovered timepoints) (Miltex Medical Biopsy Punch Dermal 8 mm, Integra Life Sciences, Princeton, NJ, USA). Biopsies were taken from the side of the neck opposite from that utilized for QITSTs on the day, and from a site at least 5 cm below the area previously used for QITSTs. The punch-biopsy site was closed with a single suture. Samples were then fixed in 10% buffered formalin solution and embedded in paraffin (FFPE) for 30 days as samples were originally intended for histology and not for RNA extraction.

### 2.5. Illumina Sequencing and Library Construction

We submitted triplicates of day −1, 5 and 39, totaling 54 formalin fixed paraffin embedded (FFPE) biopsies to the University of Florida Interdisciplinary Center for Biotechnology (ICBR) for RNA extraction, library preparation and sequencing. RNA was extracted using the RNeasy FFPE kit (Qiagen) and 100 ng of Protein-free, total RNA used for library construction employing the NEBNext Ultra Directional RNA Library Prep Kit for Illumina (NEB, USA). RNA Integrity Number (RIN) quantified in the Agilent 2100 Bioanalyzer averaged 2.42 across the 54 samples (Range = 1.8 to 2.6, no significant difference by horse or day). RNA integrity measures by RIN can range from 1 to 10, and values below 3 represent degraded profiles [23,24]. RNA recovery from FFPE tissues is challenging [25], however, as the original experimental design intended only a histological examination, no other samples were available for RNA-seq. RNA quantification was done utilizing the Qubit™ RNA HS assay kit and Qubit™ 3.0 fluorometer.

Libraries were constructed with 100 ng of protein-free total RNA using the NEBNext Ultra Directional RNA Library Prep Kit for Illumina (New England Biolabs [NEB], catalog #E7420—NEB, USA). Two ul of 1:2000 diluted External RNA Controls Consortium (ERCC) controls were added to the 100 ng of total RNA and followed by mRNA capture using NEBNext Poly(A) mRNA Magnetic Isolation module (NEB, catalog #E7490). Afterward, the RNA library was constructed using the NEBNext Ultra II Directional Library Prep (NEB, catalog #E7760). Finally, the library was amplified and purified with AMPure beads (Beckman Coulter, catalog #A63881). The library size and mass were assessed by the Agilent 2200 TapeStation using a DNA5000 Screen Tape. The library functionality was validated through quantitative PCR, using the KAPA library quantification kit (Kapa Biosystems, catalog number: KK4824) and monitored on the BioRad CFX 96 real-time PCR system. 

**Figure 4:**
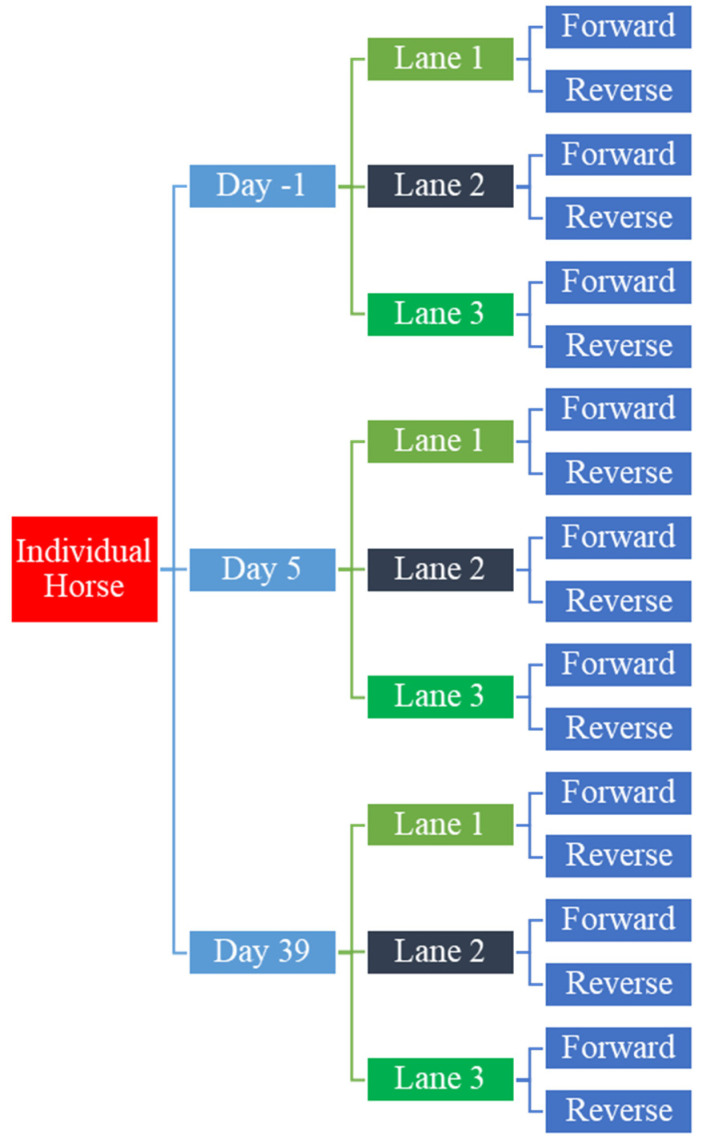
RNA-seq read analysis workflow representing how timepoint samples per individual horse were divided in 3 lanes and paired-end read.

Individual samples were pooled equimolarly at 2.5 nM. This “working pool” was used as input in the HiSeq3000 instrument sample preparation protocol (Illumina Material #20015630, Document #15066496 v04, January 2017). Eighteen RNA-Seq barcoded libraries were pooled and sequenced in multiplex on a single flow cell, using a 2 × 100 cycles (paired-end) configuration. Such sequencing configuration was achieved by pooling the reagents from a 150-cycles and a 50-cycles Illumina HiSeq3000 SBS kits. Sequencing was performed on three lanes producing 108 individual files (Figure 4). Raw sequencing files went through internal quality controls, trimmed for adaptor sequences, and delivered as fastq files (Supplemental Table S1).

### 2.6. RNA-seq Read Quality Control and Mapping

Sixty-seven (62%) of the resulting sample sequencing files had a failure to sequence percentage ranging from 18–27%. Remaining files sequenced with a failure rate of 9%. Still, sequence yields ranged from 45,284,391 to 67,875,768 reads per sample, at least 3× more than the minimal range (14–20 million) deemed acceptable for FFPE samples (Supplemental Table S1) [25]. The relatively short average read length of 101 bp and high percentage of failure within reads is likely a detrimental result of the formalin treatment used to preserve these samples and not due to faulty isolation technique [26]. We performed quality control (QC) for the raw sequence reads on FastQC v.0.11.7 [27] and visualized these results on MultiQC [28]. Utilizing kallisto [29], we aligned paired reads to the ENSEMBL release version 99 (ftp://ftp.ensembl.org/pub/release-99/fasta/equus_caballus/cdna/ (accessed on 6 September 2020)) cDNA reference [30], using the --bias flag with 100 bootstraps. Kallisto successfully aligned between 492,541 and 3,605,590 reads per individual sample/day (mean: 1,450,234) to locations in the ENSEMBL reference. Kallisto pseudo-alignment resulted in individual aligned sequences averaging 101 bp/strand, confirming the expected sizes for RNA fragments following adaptor trimming. 

### 2.7. Differential Expression Analysis

A differential expression (DE) analysis was performed using the kallisto calculated counts per million and EdgeR tools in R [31,32]. We normalized and scaled counts from kallisto-generated abundance files for length. Effective libraries generated from scaled counts accounted for individual sample composition bias and we dropped transcripts that did not have at least five estimated counts in at least 25% of samples from the analysis. A total of 22,111 transcripts were expressed in the skin samples, and further assessed for differential expression in EdgeR [33]. A principal component analysis (PCA) plot and ANOVA analyses of the PC components vs. horse or day showed no significant effect of individual horse, or day (Supplemental Figures S1 and S2).

We fitted a generalized log-linear model (GLM) to the individual foal and condition. Afterwards, we applied a Quasi-Likelihood F-test on the fitted GLM, comparing normal sweating timepoints (day −1 and day 39) versus anhidrotic (following erythromycin treatment—day 5) [33,34]. 

### 2.8. Differentially Expressed Gene Ontology and Literature Search

After Bonferroni correction for multiple testing, we found 132 differentially expressed transcripts (*p* < 2.26 × 10^−6^) (Table 1). This list of 132 genes were overlaid onto the gene ontology knowledge base using the homologous human gene symbols determined by ENSEMBL and ClueGO v2.5.7 on Cytoscape [35,36,37]. We performed a broad search in four databases: WikiPathways, CLINVAR, REACTOME and GO Biological Process, utilizing *Homo sapiens* as base organism for the gene functional annotations. Parameters set for the search included a two-sided hypergeometric test for enrichment/depletion and were multiple-test corrected by Benjamini-Hochberg. Cluster thresholds for pathway selection/GO term included a minimum threshold of two genes, representing a 2% minimum percentage of number of genes in each pathway. We also performed a comprehensive web-search on Google Scholar and PubMed of scientific literature on significantly DE genes for terminology and symptomatology related to anhidrosis, skin conditions, macrolide drugs and gene function.

## 3. Results

### 3.1. Erythromycin Causes Hyperthermia and Diarrhea

While in stalls, three foals had rectal temperatures above 39.4 °C (103 °F) on a total of five occasions. Hyperthermic foals were cared for as described in Materials and Methods until their body temperature was below 38.9 °C. Two foals had diarrheal episodes, which were mild and self-limiting. One subject required administration of nonsteroidal anti-inflammatory drugs for mild signs of colic. 

### 3.2. Thermoregulatory Sweat Response Is Impaired by Erythromycin

**Figure 5:**
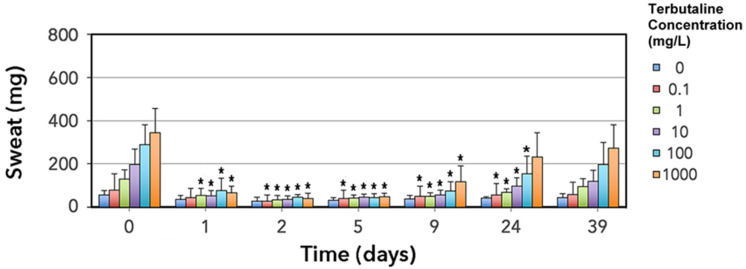
Weights (mean ± SD) of sweat collected into absorbent pads over intradermal saline or terbutaline injections in 6 foals treated with erythromycin. For each terbutaline concentration, days where sweat weights significantly differ to baseline (pooled and averaged data from day −2, −1 and 0) are marked with an asterisk (*).

Erythromycin administration significantly changed terbutaline sweat responses (*p* < 0.0005; 2- factor day × terbutaline concentration repeated measures interactions). Compared to baseline (days −2, −1, 0), sweat weights in foals given erythromycin were significantly lower (*p* < 0.05) on all treatment days (1, 2), and on post-treatment days 5, 9 and 24. By day 39, sweat weights were no longer significantly different from their pre-treatment values, indicating that the foals recovered the ability to sweat (Figure 5).

### 3.3. Differentially Expressed Genes Are Involved in Mitosis, Ion Channel Function, Ubiquitin Activity, and Immune Response

Of the 22,111 expressed transcripts in our dataset, Bonferroni correction for multiple testing detected 132 that are differentially expressed (*p* ≤ 2.26 × 10^−6^) between anhidrotic and normal sweating response (baseline plus recovered) timepoints. Of this significant group of transcripts, 114 were upregulated, and 18 downregulated. Fourteen (10.6%) of the 132 total transcripts have no assigned identification (ID) symbol or annotation associated in the EquCab3.0 reference genome, only ENSEMBL stable ID prefixes, thus only 118 transcripts have annotated gene IDs. 

**Figure 6:**
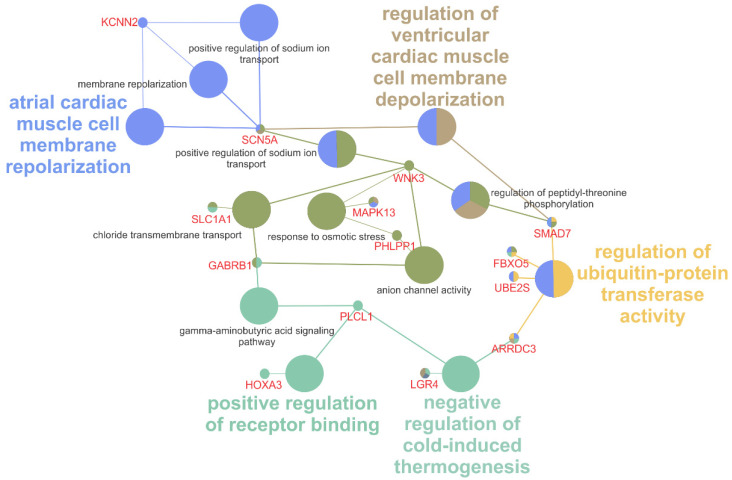
Graphical representation of the ClueGO significant terms that share a background in ion channel function and are correlated to erythromycin side effects. Groups are demonstrated by the same color, and grey-labeled processes represent overrepresented minor processes within larger groups (colored labels).

ClueGO analysis of the DE genes resulted in identification of over-represented ontologies relevant to macrolide-induced anhidrosis including *negative regulation of cold-induced thermogenesis* (GO:0120163), *response to osmotic stress* (GO:0006970), *cardiac muscle cell membrane repolarization* (GO:0099622), *anion channel activity* (GO:0005253), *chloride transmembrane transport* (GO:1902476) and *positive regulation of sodium ion transport* (GO:0010765). Some ontology terms were also related to other side-effects of macrolide administration, including *microvillus organization* (GO:0032528) and *regulation of ubiquitin activity* (GO:0051444) (Figure 6). The antimicrobial, anti-inflammatory and immune-modulating activity of erythromycin itself [38] can explain the detection of biological processes related to immunity and antibiotic treatment: *negative regulation of adaptive immune response based on somatic recombination of immune receptors built from immunoglobulin superfamily domains* (GO:0002823), *negative regulation of lymphocyte mediated immunity* (GO:0002707) and *negative regulation of T cell activation/differentiation* (GO:0050868, GO:0045581). 

The DE genes Potassium Calcium-Activated Channel Subfamily N Member 2 (*KCNN2*), Solute Carrier Family 1 Member 1 (*SLC1A1*) and Sodium Voltage-Gated Channel Alpha Subunit 5 (*SCN5A*) are highlighted by significantly overrepresented GO terms (Figure 6). These three genes are involved in cross-membrane ion transport. *KCNN2* encodes SK2, a protein that forms a voltage-independent calcium (Ca^2+^)-activated channel [39]. *SCN5A* encodes a tetrodotoxin-resistant voltage-gated sodium (Na^+^) channel subunit that mediates Na^+^ voltage-dependent permeability of membranes, forming a selective channel that functions in response to the cellular ion gradient [40]. In humans, variants in these genes are implicated in cardiac pathologies including arrythmias and fibrillations [39,41,42,43]. *SLC1A1* is an essential high-affinity glutamate transporter with a role in transporting one amino acid molecule together with a sodium ion, while counter-transporting a potassium (K^+^) ion. This gene is expressed in skin and sweat glands in humans, according to the Gene ORGANizer database [44]. *SLC1A1* variants are commonly implicated in glutamargic-related neurological abnormalities like Obsessive-Compulsive Disorder [45] and or sensory epilepsy due to contact with hot water [46].

## 4. Discussion

Despite an increase in drug resistance in recent years, macrolides are still the treatment of choice for Rhodococcus infections in foals [47,48]. Although a previously documented side-effect of erythromycin administration, the etiology of sweat-suppression by this drug is not well understood, despite its potentially lethal consequences [19,20]. As expected, sweat response in this study was significantly altered during erythromycin treatment in healthy foals (*p* < 0.0005), and diarrheal episodes were observed [9,19]. 

Although RNA sampling was not included in the original study design, the opportunity to use a hypothesis-generating approach like RNA-seq to better characterize a poorly understood condition like anhidrosis was pertinent to the posed hypothesis. Due to RNA degradation (illustrated by relatively low RIN values) we normalized and scaled read counts, as well as accounting for individual sample composition bias and we selectively ignored under-observed transcripts with less than five estimated counts in at least 25% of samples. We have also applied strict thresholds in multiple QC steps and appropriate multiple-testing corrections for data analysis and results. This “over-conservative” approach was intended to minimize false positive outcomes from low quality samples. Thus, these findings will illuminate novel potential mechanisms behind this condition and future research should include validation of these findings by additional targeted techniques like qPCR and immunohistochemistry. 

Significant DE genes within overrepresented GO terms are likely relevant to the diverse consequences of erythromycin administration, in addition to transient anhidrosis. Four genes participate in pathways for ubiquination and its regulatory components. Ubiquitin is a family of proteins involved in a plethora of biological functions, ranging from cell differentiation to adaptive immunity [49]. Specifically, SMAD Family Member 7 (*SMAD7*) was upregulated 5.71-fold during erythromycin-induced anhidrosis. Overexpression of *SMAD7* in skin decreases hair follicle size, accelerates development of sebaceous glands, as well as increases β-catenin degradation by ubiquitination-related factor in carcinogenesis and other severe pathologies of the epithelial tissue [50,51]. *SMAD7* downregulation on transforming growth factor β (TGFβ) leads to endogenous skin inflammation [52]. The reported upregulation of this gene could be due to the aforementioned immune-modulating, antimicrobial, and anti-inflammatory activity of erythromycin [38] or to an inflammatory response of the organism to erythromycin-induced cellular lesions.

Erythromycin antimicrobial effects may also be reflected in the DE gene set: centrosomal protein 85 (*CEP85*), F-box protein 5 (*FBX05*), HAUS augmin like complex subunit 1 (*HAUS1*), large tumor suppressor kinase 2 (*LATS2*), mitogen-activated protein kinase 13 (*MAPK13*) and RHO family interacting cell polarization regulator 2 (*RIPOR2*). These genes are involved in the cellular cycle by regulating mitosis [53] and overexpression may lead to impairment of cell division. This cell cycle impairment mechanism is caused by erythromycin, as part of the antibacterial/microbial effect of this drug [54]. Nevertheless, the mitotic impairment is not restricted to bacteria, also inhibiting division of rat spermatids and fibroblast-like human cells [55,56]. 

### 4.1. Erythromycin Activation of SK2 Channel as a Cause of Diarrhea

Though often mild and self-limiting, diarrhea can be fatal to the foal, an effect often attributed to intestinal bacteria dysbiosis [9,57,58,59]. In humans, macrolide treatment of bacterial infections has no known adverse effect on the sweat response, but can result in stimulation of gastrointestinal (GI) movement and diarrhea [60,61]. Erythromycin itself is a motilin receptor agonist and stimulates intestinal motor activity through activation of calcium channels [62,63]. The DE gene *KCNN2* contributes to the regulation of GI tract smooth musculature excitability [64]. This small conductance Ca^2+^-activated K^+^ channel (SK) gene is highly expressed in the gastrointestinal tract musculature of several mammal species [64]. Although overexpression of *KCNN2* in this study was noted in skin, a parallel effect in GI tissues due to erythromycin administration is a plausible hypothesis for the secondary non-fatal diarrhea.

### 4.2. Upregulation of Ion Channels and β2-Adrenergic Receptors by Erythromycin in Thermoregulatory Sweat

In horses, sweat secretion is hypertonic relative to plasma in Na^+^, Cl^−^ and K^+^ [65]. The concentration of K^+^ and Cl^−^ ions in sweat from chronic anhidrotic adult horses is significantly higher than normally sweating individuals, increasing respectively by 2.7- and 1.8-fold [66]. The upregulation of genes involved in Cl^−^ and K^+^ transport following erythromycin administration corroborates prior hypotheses of suggesting altered function of ion channels leading to anhidrosis [19,20]. Macrolides, including erythromycin and clarithromycin, acutely decrease the Cl^−^ secretion of epithelial cells [67]. Upregulation of ion channel related genes in response to erythromycin may be a compensatory response intended to reestablish normal sweat function in the event of damage or impairment of these ion channels.

In vitro overexpression of *SLC1A1* in human neuroblastoma SK-N-SH cells is correlated with decreased cytokine expression after an oxidative inflammatory challenge [68]. Macrolides decrease cytokine production especially in airway diseases, where cytokines are crucial inflammatory response regulators. In human Cystic Fibrosis (CF) patients, administration of the macrolide azithromycin decreased systemic inflammation and slightly improved (5.4%) forced expiratory volume in one second (FEV_1_), slowing lung function deterioration [69,70,71]. CF is a channelopathy caused by mutations in the cystic fibrosis transmembrane conductance regulator (*CFTR*) gene, impairing Cl^-^ channel function [72]. It is primarily characterized by the progressive loss of lung function and extreme inflammatory response of airway cells, even in the absence of an infecting agent [73,74,75,76]. Although connected to inflammatory response and in consequence to a channelopathy, this ion channel gene overexpression may be an effect of the anti-inflammatory component of erythromycin, rather than a cause of transient anhidrosis. 

One of the DE ion channel genes, expression of *KCNN2* in human skin is uniquely limited to melanocytes, and not observed in keratinocytes and fibroblasts [77]. Human thermoregulatory sweat glands are eccrine in type, possessing no direct association with hair follicles or melanocytes [78]. Although equine sweat glands are eccrine in function [16], their serpentine excretory ducts are closely associated with hair follicles and melanocytes, a characteristic observed in apocrine-type glands [79,80]. The *KCNN2* gene codes for the SK2 protein, a potassium channel. Ten-fold overexpression of the SK2 protein in mutant mice (SK2+/T, *Kcnn2^tm2Jpad^*) leads to elevated channel-mediated restriction of glutamatergic excitatory postsynaptic potential [81]. The SK2 channels are voltage independent and activated by intracellular Ca^2+^, and overexpression increases the blockage to Ca^2+^ influx [81]. Calcium is also an essential intracellular messenger for purinoreceptor-mediated sweat in horses [82]. The impaired calcium influx could lead to accumulation of K^+^ ions within the sweat-gland lumen, increasing extracellular/sweat K^+^ respective concentration in anhidrotic individuals [83]. A similar increase in relative sweat K^+^ concentration is observed in sweat from chronic anhidrotic adult horses [66,83]. 

Oral usage of erythromycin in humans is associated with prolonged cardiac repolarization times, and two-fold higher risk of sudden death from a cardiac event [84]. Upregulated in foals following erythromycin treatment, the *SCN5A* gene encodes the Na_v_1.5 channel, responsible for cardiac action potential through influx of sodium ions [40,85]. Human *SCN5A* transgene TG-WT mouse hearts with approximately 10 copies of the transgene (TG-WT L10) demonstrated premature atrial contractions, as well as shortened PR interval and P wave duration [86]. Although overexpression altered depolarization measurements, no other abnormalities (e.g., arrythmias) were noted, therefore overexpression of *SCN5A* does not seem to cause any severe cardiac pathologies [86]. Nevertheless, loss of function mutations in a gene family member: *SCN9A*, the gene encoding sodium channel α-subunit Na_v_1.7, lead to channelopathy-associated insensitivity to pain, with a subset of these patients also presenting anhidrosis [87,88]. Functional consequences of overexpression or even functional variants of the *SCN5A* gene are unknown in the horse.

Upregulated 6.741-fold during transient erythromycin-induced anhidrosis, the arrestin domain containing 3 (*ARRDC3*) gene downregulates β_2_-adrenergic receptors (β_2_-AR) by promoting retention of early endosomes and delaying recycling. *ARRDC3* also mediates cAMP production by β_2_-AR-dependent modulation [89] while epinephrine stimulates β_2_-AR [90]. Sweating in horses is dependent upon the actions of circulating epinephrine [91,92] and equine sweat glands are primarily under β-adrenergic control [93]. The involvement of β-adrenoreceptor pathways could be one trigger for equine idiopathic anhidrosis [16,83,91,92]. Appropriate downregulation of β_2_-AR is crucial for normal function of diverse biological processes, as it acts in an anti-regulatory mechanism through ubiquination [94]. Still, erythromycin can limit the drug efficacy of β_2_-agonists by downregulation of β_2_-AR in treatment of human asthma, a mechanism known as tachyphylaxis (loss of efficacy following dosing) [95]. In horses, a failure in β-adrenergic stimulation can lead to impairment of epithelial ion transport [96]. Erythromycin administration in foals could lead to a blocking effect by upregulating *ARRDC3*, thus retaining cAMP and β_2_-AR interacting endosomes, leading to a transient lack of response to thermal stimuli and to the β_2_-agonist terbutaline, utilized in the QITST test. 

## 5. Conclusions

While RNA recovery from FFPE tissue yielded relatively few high-quality mapped reads, we were still able to utilize this unique data set to investigate differential gene expression. Previously hypothesized involvement of ion channels and β_2_-adrenergic receptors in the development of erythromycin-caused transient anhidrosis was confirmed by identification of genes relevant to these functions among those differentially expressed in this experiment. We also identified differentially expressed genes involved in other biological mechanisms of erythromycin treatment, including antimicrobial, anti-inflammatory, and immune-modulating effects. Differential expression of genes involved in ion channels and β_2_-adrenergic receptors demonstrates a possible correlation between transient anhidrosis secondary to erythromycin treatment and Chronic Idiopathic Anhidrosis in the horse. Our findings suggest potential targets for further investigation for therapeutic intervention, including *KCNN2*, *SC5NA* and *ARRDC3*. This first step into differential expression analysis will pave the way for the development of therapeutic agents that could reduce or eliminate negative side-effects of macrolide drugs in foals.

## Figures and Tables

**Table 1:** Top 10 significant downregulated and upregulated differentially expressed genes (the full list can be found in Table S2).

	Gene Symbol	logFC	logCPM	LR	*p* Value
DOWNREGULATED	*ENSECAT00000046497*	−7.38989	7.370481	38.36492	7.22 × 10^−8^
	*MAP3K1*	−7.90642	6.997842	40.21128	1.69 × 10^−7^
	*ATP5F1E*	−8.13404	6.513773	39.98864	1.79 × 10^−7^
	*ENSECAT00000003591*	−6.44825	6.857703	34.98519	2.01 × 10^−7^
	*CXXC1*	−6.81169	6.772696	34.79323	2.19 × 10^−7^
	*CTNNBL1*	−6.88469	6.306215	37.67177	5.04 × 10^−7^
	*ENSECAT00000004781*	−5.20358	5.421982	37.01026	5.81 × 10^−7^
	*BTAF1*	−6.27921	7.804846	31.50879	6.34 × 10^−7^
	*DRG1*	−6.94174	6.382133	32.17161	7.78 × 10^−7^
	*MEST*	−7.69547	7.025976	34.08881	8.49 × 10^−7^
UPREGULATED	*HAUS1*	7.426906	5.73278	153.6852	1.68 × 10^−15^
	*SMPD4*	6.856443	6.812866	110.1973	2.86 × 10^−14^
	*ARRDC3*	6.410874	6.280866	87.45059	9.47 × 10^−12^
	*PLCL1*	6.732944	5.847365	78.57107	4.21 × 10^−11^
	*FBXO5*	4.928078	6.33902	72.20562	1.32 × 10^−10^
	*SMAD7*	5.714715	5.256898	79.97352	2.28 × 10^−10^
	*TRAK2*	5.459412	5.220843	79.77119	2.35 × 10^−10^
	*KLF5*	6.298051	6.073846	60.85152	4.25 × 10^−10^
	*REV1*	4.162814	4.400905	64.81132	5.39 × 10^−10^
	*GABRB1*	5.821451	5.342432	68.36093	1.41 × 10^−9^

## Data Availability

The transcriptomics dataset and sample details are hosted at NCBI Sequence Read Archive (SRA) repository and Bioproject: PRJNA649594.

## Supplementary Materials

The following are available online at https://www.mdpi.com/article/10.3390/ani11123379/s1, Table S1: Sample demographics and Illumina RNA-seq statistics, Table S2: Full set of gene differential expression, Figures S1 and S2: PCA plots and ANOVA analyses of the PC components vs. horse or day.
