# Supplementary material for: Ion Channel and Ubiquitin Differential Expression during Erythromycin-Induced Anhidrosis in Foals
Source: Animals (Basel). 2021 Nov 25;11(12):3379. doi: 10.3390/ani11123379 (PMC8697959; doi:10.3390/ani11123379)

Supplemental Figure 1. Three dimensional scatterplot of principal components 1, 2 and 3.

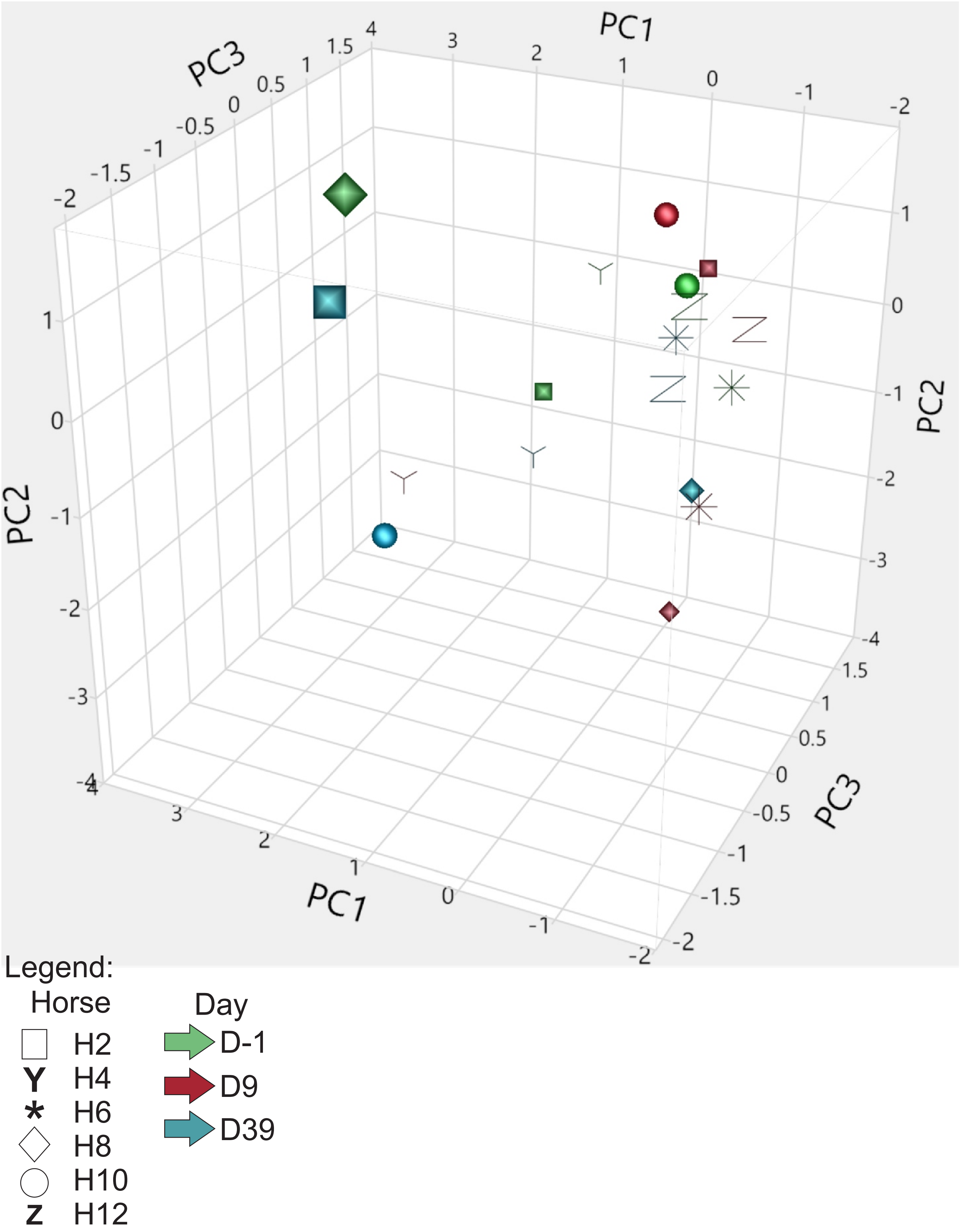

Supplemental Figure 2. ANOVA analyses of the principal components 1 (a, b), 2 (c, d), and 3 (e, f) versus horse (a, c, e) or day (b, d, f) show no significant effect of individual horse or day ($p > 0.05$). Respective significance values are noted in each plot.

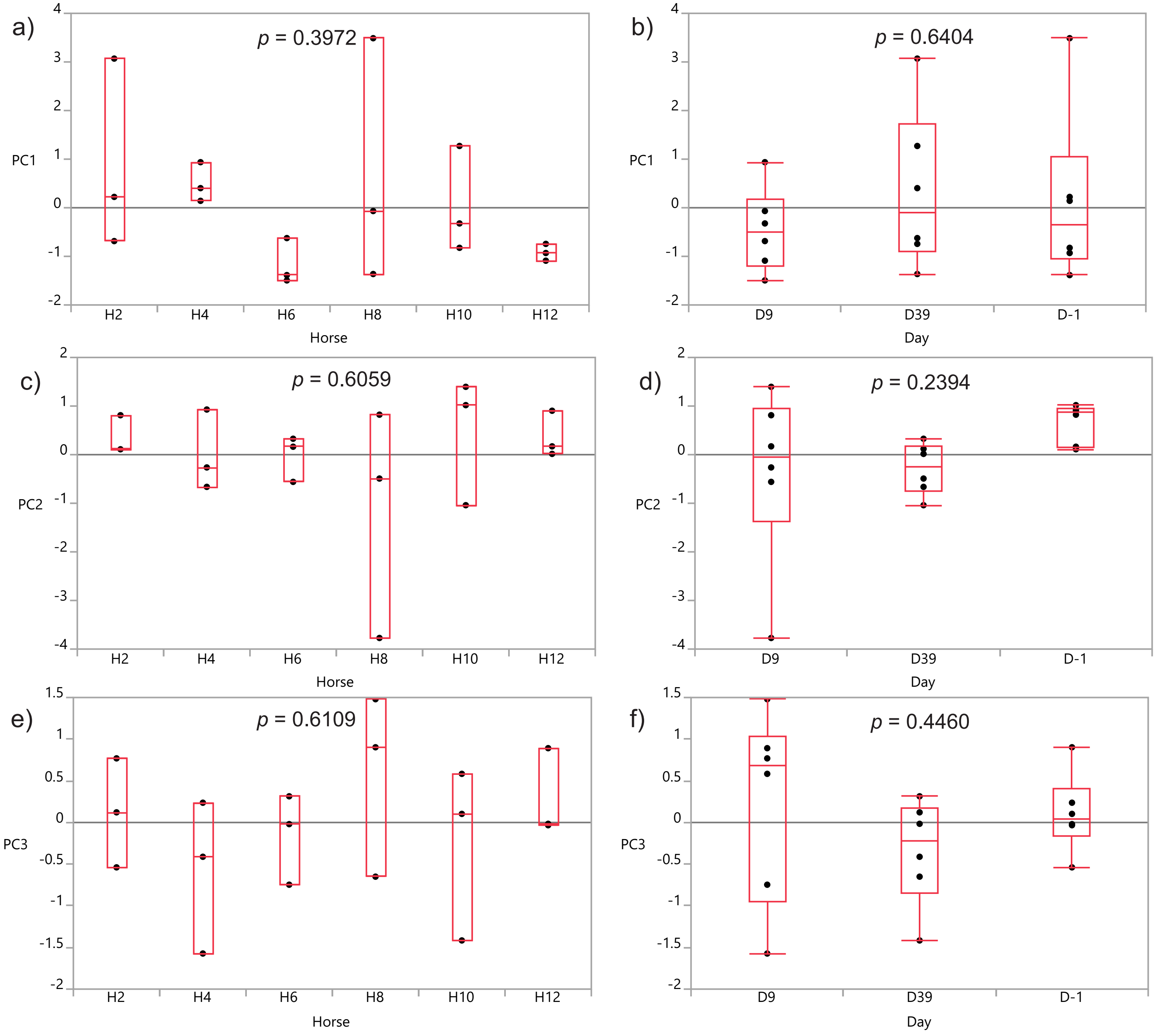

Supplement: Supplementary file 1 [file animals-11-03379-s001.zip › Supplemental Figures 1-2.pdf]
